# Time to initial highly active antiretroviral therapy discontinuation and its predictors among HIV patients in Felege Hiwot comprehensive specialized hospital: a retrospective cohort study

**DOI:** 10.1186/s12981-021-00418-z

**Published:** 2021-12-04

**Authors:** Tewodros Getnet Amera, Kassawmar Angaw Bogale, Yibekal Manaye Tefera

**Affiliations:** 1 Epidemiology and Biostatistics Department, School of Public Health, College of Medicine and Health Sciences, Dire Dawa University, Dire Dawa, Ethiopia; 2 Epidemiology and Biostatistics Department, School of Public Health, College of Medicine and Health Sciences, Bahir Dar University, Bahir Dar, Ethiopia

**Keywords:** Initial HAART discontinuation, Survival time, HIV, Bahir Dar, Ethiopia

## Abstract

**Background:**

Anti-retroviral therapy regimen discontinuations become a big challenge and cause diminishing the clinical and immunological benefit of treatment in Ethiopia. It reduces both the duration and the chance of viral control due to cross-resistance between different alternative drugs and overlapping toxicity between and within a class of antiretroviral drugs in Ethiopia. However, information’s on the time of initial regimen discontinuation and its predictors are not well studied.

**Objective:**

This study aimed to assess the time to initial highly active antiretroviral therapy discontinuation and its predictors among HIV patients in Felege Hiwot comprehensive specialized hospital, North West Ethiopia.

**Method:**

Institution-based retrospective cohort study was conducted among 418 HIV patients who started HAART from January 1, 2014, to December 31, 2019. Data were collected from the patient chart using a data extraction tool. The Kaplan–Meier curve was employed to compare survival rates. Multivariable Cox proportional hazard regression was applied to identify independent predictors of time to initial regimen discontinuation.

**Result:**

A total of 418 patients on anti-retroviral therapy were followed. Incidence of initial HAART discontinuation was 16.7/100 person year. The median survival time was 3.5 years. Predictors showed association for time to initial HAART discontinuation were taking > 1 ART pills/day (AHR = 4.1, 95% CI 3.0–6.5), baseline CD4 count < 100 cells/mm^3^ (AHR = 2.6, 95% CI 1.5–4.7), 100–199 cells/mm^3^ (AHR = 2.2, 95% CI 1.2–4.0), baseline WHO clinical stage IV (AHR = 2.68, 95% CI 1.6–4.3) and stage III (AHR = 2.6, 95% CI 1.4–4.3) and TB infection (AHR = 2.3, 95% CI 1.6–3.5).

**Conclusion:**

Most of the discontinuation occurred after 1 year of initiation of HAART. Baseline WHO clinical stage, TB infection, baseline CD4 count, and taking > 1 ART pill/day were found predictors of initial HAART regimen discontinuation. Work on early detection of HIV before the disease is advanced and initiation of one ART regimen daily is vital for survival on the initial regimen.

## Background

Proper management of patients living with the human immune deficiency virus (HIV) is a comprehensive, lifelong process focused on the patient’s needs. The core component of treatment and care of people living with HIV is the provision of antiretroviral treatment. The optimal form, known as "highly active antiretroviral treatment" (HAART), increases the length and quality of life for infected individuals while reducing the onward transmission of the virus [1].

HAART, or simply antiretroviral therapy (ART), is the use of a combination of three or more antiretrovirals to achieve durable suppression of viral replication. Since the evolution of the first agent, Zidovudine, in 1987, substantial advances have been made in ART. Only patients with advanced symptomatic disease were prescribed Zidovudine as a monotherapy in a five-times-daily dose. In mid-1996, it was discovered that these drugs are far more effective when three or more are taken at the same time. The advent of these highly active antiretroviral treatments has dramatically reduced the morbidity and mortality associated with HIV infection and has improved the prognosis for people living with HIV infection (PLHIV) or AIDS [2, 3].

These optimum clinical and public health achievements of antiretroviral therapy require consistent long-term adherence. HIV/AIDS is a chronic, lifelong disease with no known cure, and therefore, people living with HIV have to be followed medically for the rest of their lives [4, 5]. However, currently, initial HAART regimen discontinuations become a big challenge and cause diminished clinical and immunological benefits of treatment. It also reduces both the duration and the chance of viral control due to cross-resistance between different alternative drugs and overlapping toxicity between and within a class of antiretroviral drugs [6, 7]. Second-line ART is also more expensive than first-line ART, and frequent switching may exhaust future options for effective treatment and increase mortality and morbidity due to HIV/AIDS [8–10]. In addition, discontinuation in treatment and poor adherence limit the therapeutic success of the initial regimen and sustainability of the HIV treatment program since antiretroviral therapy is a lifelong therapy [9].

As long as there is virological success, the initial regimen continues unless there is a need for regimen discontinuation due to drug side effects, co-morbid illness, pregnancy, and other conditions [8, 10, 11]. However, a significant proportion of the patients who begin HAART experience discontinuation of the initial HAART regimen and switch to another regimen, as different observational studies showed [8, 12–14]. A multi-center study in North America and Europe showed that the incidence of initial regimen discontinuation was 14.4 persons per 100 persons per year (PY) [15] and that the incidence of regimen discontinuation in Ethiopia was 16.08/100 PY [16].

Different studies showed that baseline WHO clinical stage [8], treatment failure/virological failure [13, 17], HIV/TB co-infection [8], and drug unavailability were some predictors of initial regimen discontinuation.

In resource-limited settings, including Ethiopia, where treatment options are limited, designing strategies to increase the durability of the original regimen is essential. To achieve this goal, it is important to determine the time when the initial HAART is discontinued and predictors for the time to initial HAART regimen discontinuation. However, information on the time to initial HAART regimen discontinuation and its predictors in Ethiopia is not well studied.

Therefore, this study is aimed at determining the time of initial HAART regimen discontinuation and its predictors among HIV patients. Results from this study will help to design appropriate measures to increase the duration of the original regimen among patients on antiretroviral therapy that preserve future treatment options.

## Method and materials

### Study design and study setting

An institution-based retrospective cohort study was conducted to assess the time of initial HAART regimen discontinuation and its predictors among HIV seropositive patients initiated on HAART at Felege Hiwot comprehensive specialized hospital, Bahir Dar, North West Ethiopia, from January 1, 2014, to December 31, 2019.

Felege Hiwot comprehensive specialized hospital is one of the largest hospitals in Bahir Dar, which was established in 1960. The HIV care service of the hospital was initiated in 2003. Since 2003, when the hospital started ART, 13,042 patients have been enrolled in HIV care. Six thousand six hundred twenty patients were actively following their treatment, and 1538 patients started ART between January 1, 2014, and December 31, 2019. Standardized monitoring and evaluation tools and the data collection and management process were well organized and supported by the electronic database system of the hospital, which was suitable for accessing data.

### Source and study population

The source population was all HIV patients who had been on follow-up at the ART clinic of Felege Hiwot, a comprehensive specialized hospital. The study population was all HIV patients who initiated ART at Felege Hiwot comprehensive specialized hospital from January 1, 2014, to December 31, 2019, and following their chronic care and treatment at the hospital.

### Eligibility criteria

Patients who were initiated on combined ART and had a follow-up in Felege Hiwot comprehensive specialized hospital and who began ART between January 1, 2014, and December 31, 2019, were included in the study, whereas patients who were initiated on antiretroviral treatment outside of Felege Hiwot comprehensive specialized hospital and transferred in, as well as charts with incomplete demographic and clinical information, were excluded.

### Sample size determination

Using epi info version 7.2.2 and by considering the 95% confidence interval, 5% marginal error, 80% power, and 1:2 proportions, the maximum sample size was calculated by taking factors that gave the maximum sample size from the significant predictors in previous studies. The predictors which were significantly associated with the dependent variable were: co-medication with ART [1], presence of stavudine in the regimen [2], duration of the regimen for 1 year [3], and WHO clinical stage III [1] at the initiation of HAART. With this consideration, the final sample size became 418.

### Sampling techniques and procedure

Using patient charts and the ART electronic database, data were collected from Felege Hiwot, a comprehensive specialized hospital HIV care clinic. Patient chart numbers were from the electronic database as a sampling frame. From 1538 patients who started HAART from January 1, 2014, to December 31, 2019, 418 charts were selected and reviewed by a simple random sampling technique through computer-generated random numbers.

### Study variables

The dependent variable was survival time to initial HAART discontinuation (in a year).

Independent variables were categorized in to five themes: sociodemographic factors: Age (in year), Sex, marital status, educational level and occupation; clinical factors: weight, WHO clinical stage, TB co-infection and pregnancy; Patient-related factor: Adherence status, substance use and disclosure status; drug-related factors: initial HAART regimen, ARV Drug toxicity, Pill burden, and Co-medication; and immunological and virological factors: Baseline CD4 count, latest CD4 count, initial viral load and latest viral load.

### Data tools, collection and quality control

The data abstraction tool was developed from the available information on the patient records, which had first been observed, and it had three parts: sociodemographic factors; baseline clinical, laboratory, and ART information; and patient follow-up information. Three trained nurses and a supervisor were recruited for data collection along with the principal investigator. One data clerk also supported them by identifying the charts that were retrieved using the patient registration number in the database system.

The data extraction tool was pre-tested among 5% of the sample population, and one-day training was provided for data collectors and a supervisor on the objectives and relevance of the study. The supervisor managed the data collection process every day, and the principal investigator also checked completed questionnaires for completeness of information every other day. Gaps identified were immediately communicated to the supervisor and data collectors.

### Data processing and analysis

The collected data had been coded, cleaned, and entered by EpiData version 3.1 and was exported to the statistical package for social science (SPSS) version 23 for analysis. Descriptive statistics were used to describe the demographics, baseline, and follow-up data. The incidence density rate was calculated for the entire study period. To calculate the rate of regimen discontinuation among people on ART, the total duration of follow-up for the whole cohort in person-year (PY) was used.

The follow-up duration for people on ART who did not discontinue their initial regimen was calculated from the time of initiation of ART until the last visit. For patients who discontinued their initial regimen, the follow-up duration was calculated from the initiation of HAART to the substitution of at least one drug from the original regimen. Subsequently, the number of cases who discontinued their initial regimen within the cohort was divided by the total initial regimen discontinuation free follow-up duration and reported per 100 person-years.

The survival analysis technique was carried out as this study considered time-to-event data and the Cox proportional hazard model was fitted. The Kaplan–Meier curve has been used to estimate the median duration of survival with an initial regimen and to compare the survival curves. Both the bivariable and multivariable Cox proportional hazard models were used to identify the predictors. Variables with a p-value of less than 0.2 in the bivariable analysis were entered into the multivariable proportional hazard model. The hazard ratio's 95% confidence interval (CI) was calculated, and variables with a p-value of less than 0.05 in the multivariable Cox proportional hazards model were considered significantly and independently associated with the dependent variable. The Cox proportional hazards model fitness was checked using the Schoenfeld residuals test.

## Results

### Socio-demographic characteristics of the respondents

Four hundred eighteen (418) records were analyzed. The mean age at the initiation of ART was 31.9 ± 11.5 years. More than half of the respondents, 248 (59.3%), were female, and the majority, 380 (90.9%), were Orthodox Christians. The majority, 318 (76.1%), of the respondents were urban dwellers. Regarding marital status, 181 (43.3%) of respondents were married, and 122 (29.2%), 101 (24.2%) of respondents were single and divorced, respectively. Two hundred eighty-one (67.2%) of the study participants had occupation; among which 97 (23.2%), 82 (19.6), 49 (11.7%), and 53 (12.7%) were civil servants, merchants, farmers, and daily laborers respectively. A total of 193 (46.2%) patients had disclosed their HIV status to their family or other relatives. Three hundred eighty-nine of the study subjects (93.1%) did not use any substance (Table 1).

**Table 1:** Socio demographic characteristics of HIV positive patients at initiation of HAART at Felege Hiwot comprehensive specialized Hospital, January 1, 2014 to December 31, 2019

Variable	Category	Frequency	Percentage (%)
Sex	Male	170	40.7
	Female	248	59.3
Marital status	Single	122	29.2
	Married	181	43.3
	Divorced	101	24.2
	Widowed	14	3.3
Religion	Orthodox	380	90.9
	Muslim	34	8.1
	Protestant	3	0.7
	Others	1	0.2
Level of education	No formal education	104	24.9
	Primary education	140	33.5
	Secondary education	90	21.5
	College diploma and above	84	20.1
Occupation	Civil servant	97	23.2
	Merchant	82	19.6
	Farmer	49	11.7
	Daily laborer	53	12.7
	Other*	137	32.8
Residence	Urban	318	76.1
	Rural	100	23.9
Disclosure status	Disclosed	193	46.2
	Not disclosed	225	53.8
Substance use	Yes	29	6.9
	No	389	93.1

*Refers to study participants who have no regular occupation that can generate any income, like housewives, elderly people who have been pensioned off, and students

### Baseline clinical, laboratory, and ART information of the study subjects

The majority of the participants, 362 (86.6%), had working functional status, while 41 (9.8%) were ambulatory, and 15 (3.6%) were bedridden at the initiation of the initial HAART regimen. The mean weight of the participants was 51.58 ± 13.8 kg. The predominant HAART regimen initially prescribed for them was a combination of Tenofovir, Lamivudine, and Efavirenz (TDF-3TC-EFV), which accounted for 264 (63.2%) of cases, followed by Zidovudine, Lamivudine, and Efavirenz (AZT-3TC-EFV) with 72 (17.2%) cases. One hundred eighty-six (44.5%) were in WHO clinical stage I at the initiation of HAART, while 77 (18.4%), 76 (18.2%), and 79 (18.9%) were in stage two, three, and four at baseline, respectively.

Regarding baseline CD4 count, 155(37.1%) of participants had greater than 350 cells/mm^3^, followed by 97 (23.2%) within the category of 200–349 cells/mm^3^. About 307 (73.4%) of study subjects initiated HAART at the HGB level of ≥ 13 g/dl. Nearly 25.6% of the patients started by taking more than one ART pill/day, and 222 (53.1%) of the participants have taken Co-trimoxazole preventive therapy Prophylaxis. Nearly 84% of the study participants had good adherence status before initial HAART discontinuation. Eighty-three (19.9%) of study subjects had Tuberculosis infection while on ART, and nearly 28.7% of participants had co-medication history other than cotrimoxazole preventive therapy (Table 2).

**Table 2:** Baseline clinical, laboratory and ART information of HIV positive patients, at Felege Hiwot comprehensive specialized hospital from January 1, 2014 to December 2019

Variables	Category	Frequency	Percentage (%)
Initial regimen	TDF-3TC-EFV^a^	264	63.2
	TDF-3TC-NVP^b^	8	1.9
	AZT-3TC-EFV^c^	72	17.2
	AZT-3TC-NVP^d^	56	13.4
	ABC-3TC-EFV^e^	18	4.3
Number of ART pills	One pill/day	311	74.4
	> one pill/day	107	25.6
Functional status	Working	362	86.6
	Ambulatory	41	9.8
	Bedridden	15	3.6
Baseline WHO clinical stage	I	186	44.5
	II	77	18.4
	III	76	18.2
	IV	79	18.9
Baseline CD4 count	< 100 cells/mm^3^	88	21
	100–199 cells/mm^3^	78	18.7
	200–349 cells/mm^3^	97	23.2
	> = 350 cells/mm^3^	155	37.1
Adherence	Good	353	84.4
	Fair	32	7.7
	Poor	33	7.9
Past OI	Yes	36	8.6
	No	382	91.4
CPT prophylaxis	Yes	222	53.1
	No	196	46.9
Baseline hemoglobin	< 7 g/dl	1	0.2
	7–9.9 g/dl	13	3.1
	10–12.9 g/dl	97	23.2
	≥ 13 g/dl	307	73.4
Viral load	< 1000 copies/ml	366	87.6
	> = 1000 copies/ml	52	12.4
TB infection	Yes	83	19.9
	No	335	80.1
Co-medication	Yes	120	28.7
	No	298	71.3

^a^Tenofovir-Lamivudine-Efavirenz

^b^Tenofovir-Lamivudine-Nevirapine

^c^Zidovudine-Lamivudine-Efavirenz

^d^Zidovudine-Lamivudine-Nevirapine

^e^Abacavir-Lamivudine-Efavirenz

### Incidence and HAART discontinuation free survival time

A total of 418 study participants were followed retrospectively for the last 5 years, with 144 (34.4%) having an event and 274 (65.6%) being censored, with 140 (51.1%) still alive and on their initial regimen, 41 (14.9%) lost to follow up, 65 (23.8%) transferred out, and 28 (10.2%) dead. The study subjects had experienced an event in 863.3 person-years (PY) of observations.

**Fig. 1:**
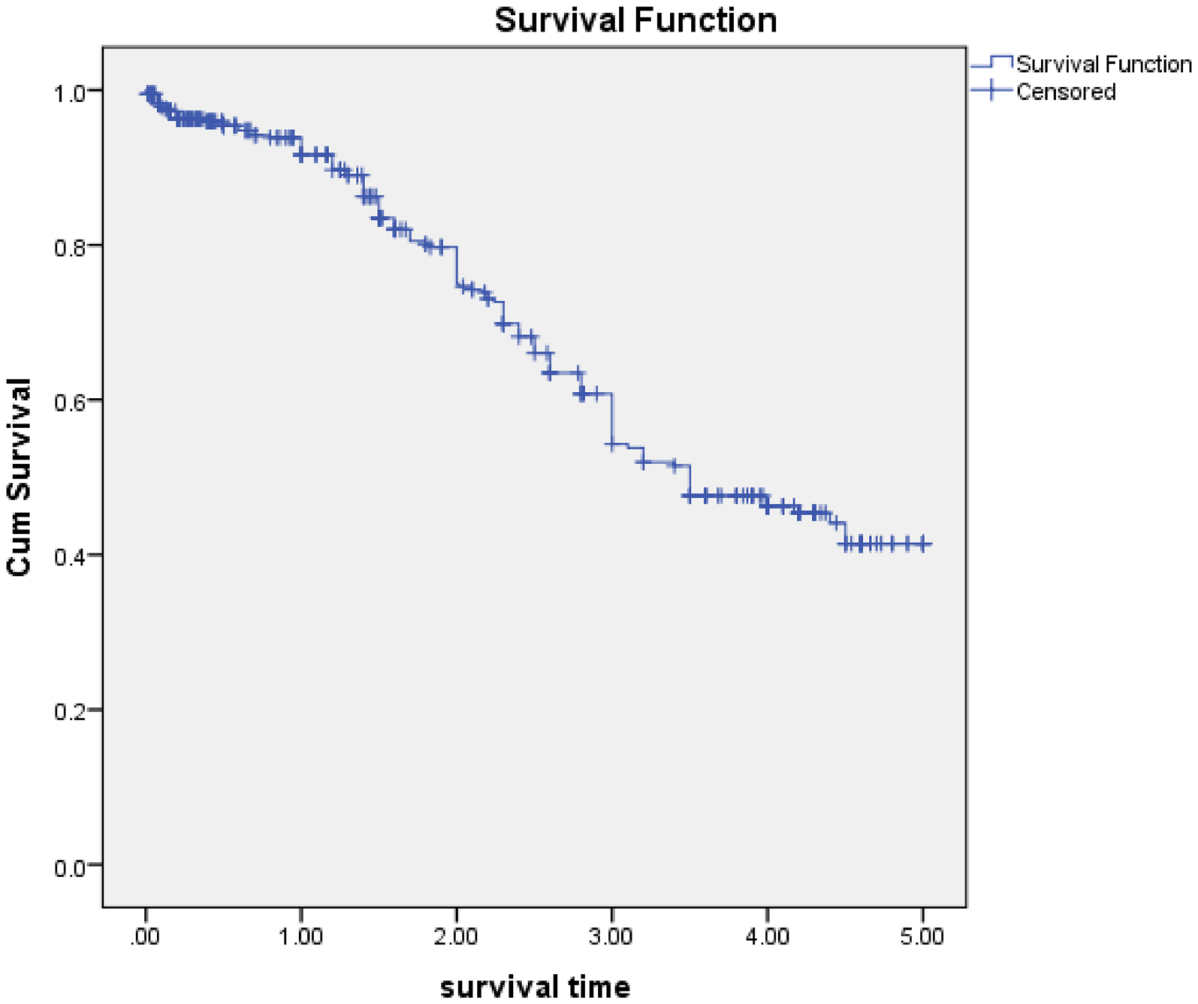
Kaplan Meier curves for time to initial HAART discontinuation among HIV patients in Felege Hiwot comprehensive specialized hospital, 2014–2019

The overall incidence rate of HAART discontinuation was 16.7/100 person-years, and the highest incidence was at second and third years. Thirty (20.8%), 45 (31.2%), 49 (34%),14 (9.7%), and 6 (4.1%) initial HAART discontinuation occurred within the first year, second years, third years, fourth years, and fifth years of the initial HAART discontinuation, respectively. The overall median survival time of the patients was 3.5 years (IQR = 2.9–4.1 years). The cumulative probability of survival without an event at one, two, three, and four years was 0.91, 0.75, 0.54, and 0.46 years, respectively (Fig. 1).

### Predictors for time to initial HAART discontinuation among HIV patients

In the bi-variable Cox-regression analysis, the following variables showed significant effects at a P-value of less than or equal to 0.2: sex, occupation, number of ART pills consumed per day, adherence, baseline CD4 count, co-medication, baseline WHO clinical stage, tuberculosis infection while on ART, and substance use showed significant effect at a P-value of less than or equal to 0.2. The other variables did not show any association with time to initial HAART discontinuation (Table 3).

**Table 3:** Cox regression analysis between different predictors and time to initial highly active antiretroviral therapy discontinuation among HIV patients in Felege hiwot comprehensive specialized hospital, northwest Ethiopia, 2014–2019

Variable		Survival status		CHR (95% CI)	P-value	AHR (95% CI)	P-value
		Event	Censored				
Sex	Male	69	101	1			
	Female	75	173	0.54 (0.38–0.74)	0.000		
Occupation	Civil servant	40	57	1			
	Merchants	29	53	0.8 (0.5–1.3)	0.39		
	Farmer	10	39	0.6 (0.32–1.3)	0.2		
	Dailey laborer	9	44	0.29 (0.14–0.61)	0.001		
	Others⃰	56	81	1.0 (0.68–1.5)	0.87		
ART pills /day	1 pill/day	79	232	1		1	
	> 1 pill/day	65	42	7.5 (5.2–10.8)	0.000	4.1 (2.7–6.1)	< 0.001
Adherence status	Good	132	235	1			
	Fair	1	15	0.19 (0.026–1.34)	0.97		
	Poor	11	24	1.3 (0.7–2.4)	0.3		
Base line CD4 count	< 100cell/mm^3^	67	21	6.6 (4.0–10.9)	0.000	2.6 (1.5–4.7)	0.001
	100–199cell/mm^3^	28	50	2.6 (1.4–4.7)	0.000	2.3 (1.3–4.3)	0.004
	200–349cell/mm^3^	29	68	2.2 (1.2–3.9)	0.08	1.7 (1.0–3.1)	0.065
	> = 350cells/m m3	20	135	1		1	
Co-medication other than CPT	No	66	232	1			
	Yes	78	42	2.7 (1.9–3.8)			
Baseline WHO clinical stage	I	29	157	1		1	
	II	32	45	2.5 (1.5–4.2)		1.2 (0.7–2.0)	0.57
	III	27	49	3.5 (1.9–5.6)		2.6 (1.5–4.5)	0.001
	Iv	56	23	6.0 (3.8–9.5)		2.68 (1.6–4.4)	< 0.001
Tuberculosis infection	No	70	265	1			
	Yes	74	9	5.3 (3.8–7.4)		2.3 (1.6–3.4)	< 0.001
Substance use	No	129	260	1			
	Yes	15	14	1.57 (0.92–2.69)			

The variables with a p-value of 0.2 or less in the bi-variable result were entered into the multivariable Cox regression analysis. In the multivariable Cox regression analysis, the number of ART pills consumed per day, baseline CD4 count, baseline WHO clinical stage, and TB infection while on ART remained significant predictors of the time to initial regimen discontinuation (Table 3).

Accordingly, the number of ART pills consumed per day showed a significant effect.

**Fig. 2:**
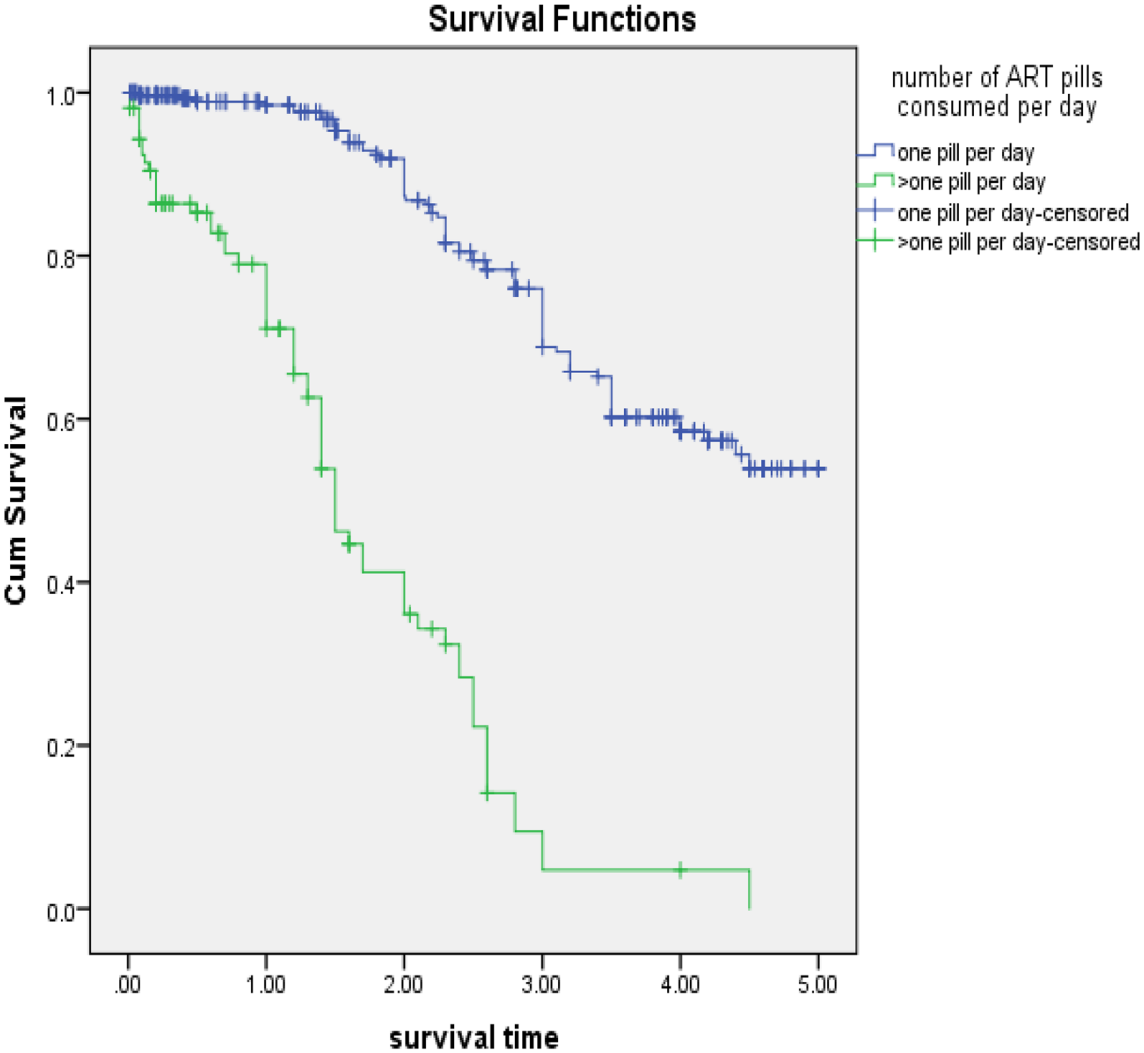
Kaplan Meier curves for time to initial HAART discontinuation among HIV patients, Felege Hiwot comprehensive specialized hospital, 2014–2019. Classification based on number of ART pills consumed per day

Patients who took > 1 pill per day had a higher risk of early initial HAART discontinuation compared to those who took 1 pill per day [p < 0.001, AHR = 4.1, 95% CI 2.7–6.1]. (Fig. 2).

**Fig. 3:**
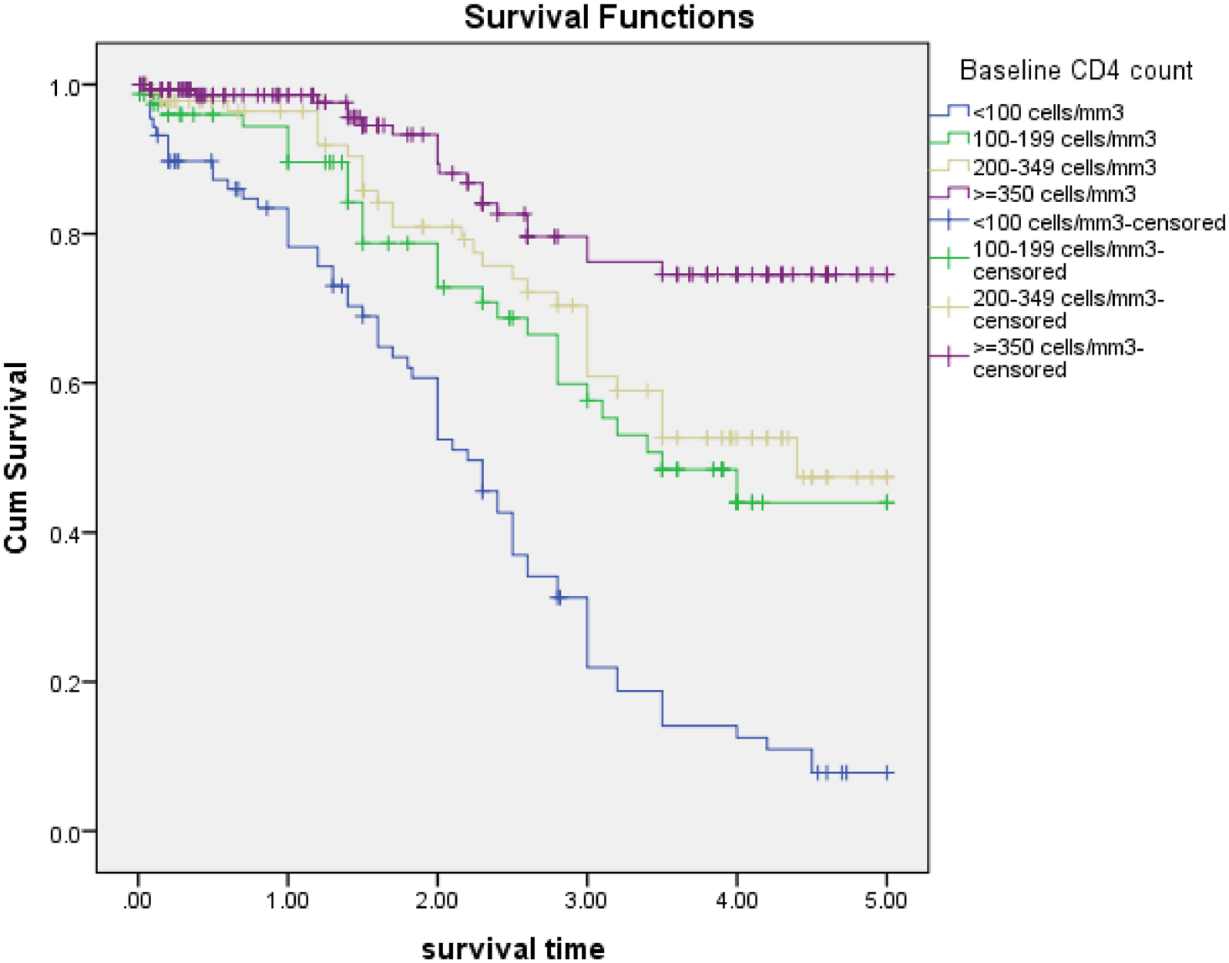
Kaplan Meier curves for time to initial HAART discontinuation among HIV patients, Felege Hiwot comprehensive specialized hospital, 2014–2019: Classification based on base line CD4 counts

The initial HAART regimen discontinuation was significantly associated with the baseline CD4 count. Those patients who had baseline CD4 count of < 100 cell/mm^3^ had higher risk for early initial HAART discontinuation when compared to those patients whose CD4 count was ≥ 350 cell/mm^3^ [p = 0.001, AHR = 2.6, 95% CI 1.5–4.7]; Patients those had 100–199 cells/mm^3^ had higher risk for early initial HAART discontinuation compared to those who had CD4 count ≥ 350 cell/mm^3^ [p ≤ 0.004, AHR = 2.3, 95% CI 1.3–4.3]; Patients those had 200–349 cell/mm^3^ had also higher risk for initial HAART discontinuation compared to those ≥ 350 cell/mm^3^ with [p = 0.065, AHR = 1.7, 95% CI 1.0–3.1] (Fig. 3).

**Fig. 4:**
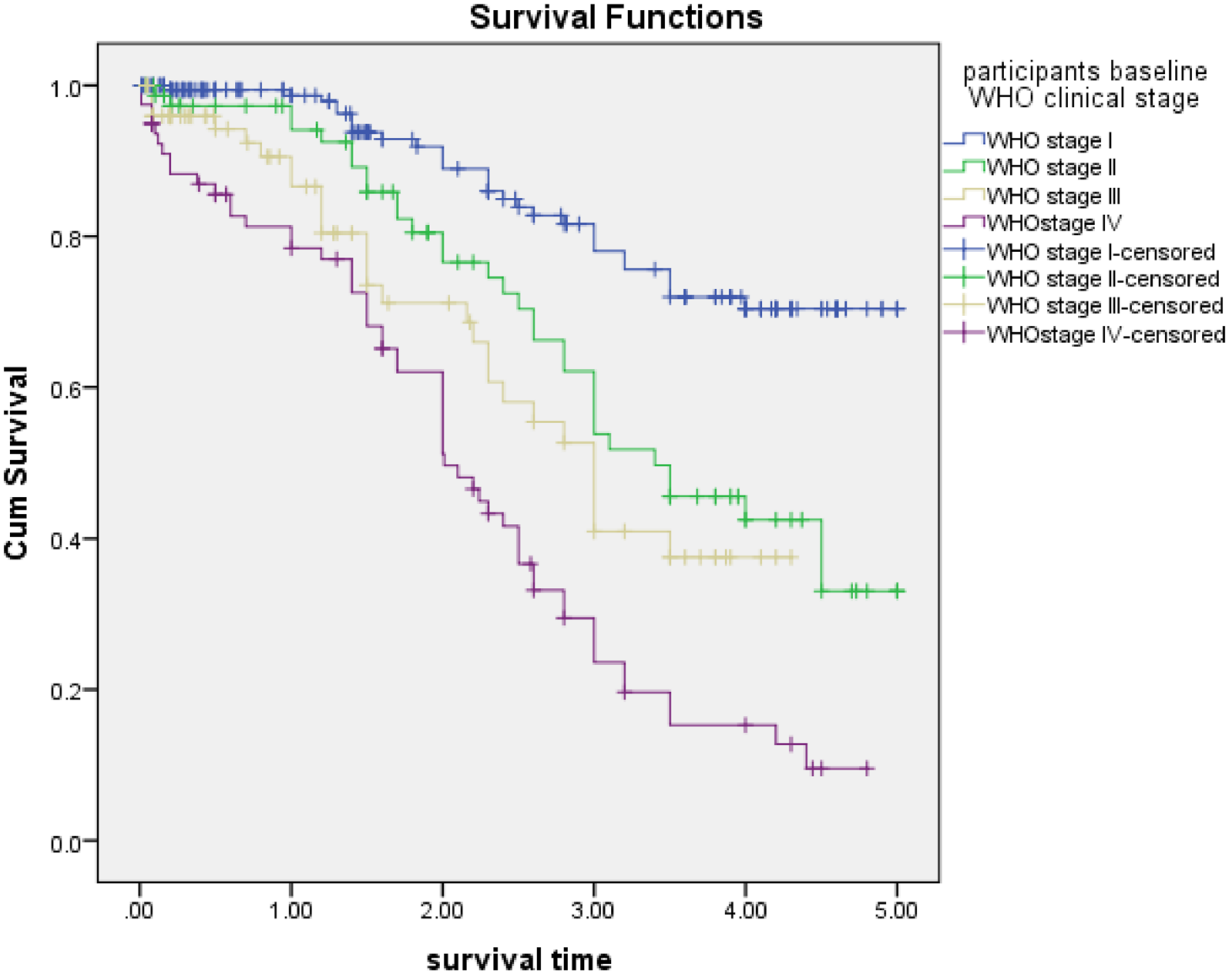
Kaplan Meier curves for time to initial HAART discontinuation among HIV patients, Felege Hiwot comprehensive specialized hospital, 2014–2019. Classification based on WHO clinical stage

HIV patients who were stage IV at the initiation of ART had a 2.68 times higher risk of early initial regimen discontinuation when compared to those who were stage I at any time [p < 0.001, AHR = 2.68, 95% CI 1.6–4.4]. Similarly, patients who had WHO clinical stage III had a higher risk of initial HAART discontinuation when compared to those who had WHO stage I [p = 0.001, AHR = 2.6, 95% CI 1.5–4.5], and patients who had WHO stage II also had a higher risk of initial HAART discontinuation compared to those who had WHO stage I [p ≤ 0.57, AHR = 1.2, 95% CI 0.7–2] (Fig. 4).

**Fig. 5:**
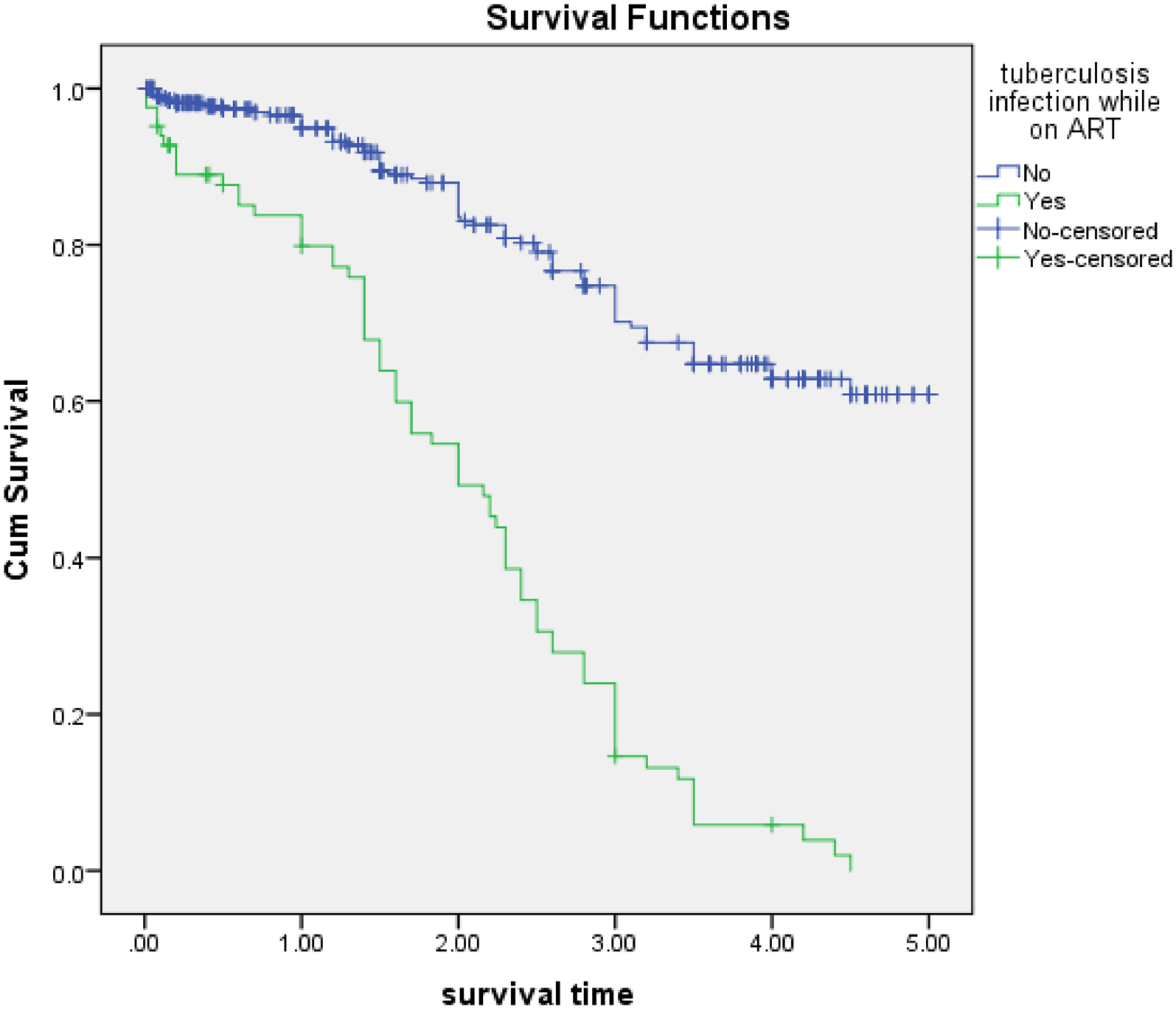
Kaplan Meier curves for time to initial HAART discontinuation among HIV patients, Felege Hiwot comprehensive specialized hospital, 2014–2019: Classification based on tuberculosis infection

Patients who developed TB on an initial regimen had a 2.3 times higher risk of discontinuing their initial regimen at any time as compared to those who did not develop TB [p < 0.001, AHR = 2.3, 95% CI 1.6–3.4] (Fig. 5).

## Discussion

The purpose of this study was to assess the time to initial HAART discontinuation and its predictors among HIV-infected patients taking ART in Felege Hiwot Comprehensive specialized hospital. Accordingly, 144 (34.4%) of the total study subjects experienced initial HAART discontinuation. From these events, 30 (20.8%) occurred within the first year, 45 (31.25%) occurred in the second year, 49 (34%) in the third year, and the remaining 14 (9.7%) and 6 (4.16%) in the fourth and fifth years, respectively. In addition, the overall incidence of initial HAART discontinuation was 16.7/100 PY.

In this study, the incidence was highest after 1 year of follow-up, which is in line with the study conducted in southwest Ethiopia, with a 24.2% and 31.2% incidence of initial HAART discontinuation in the second and third years, respectively, and northwest Ethiopia (Gondar), where the majority (90.9%) cumulative incidence of initial HAART discontinuation occurred in the third year [1, 5]. However, the study conducted in the southwest region of Cameroon indicated that the highest incidence was observed in the first year, and the study done in south India indicates that the highest incidence was observed in the first six months of follow-up [3, 6]. The difference between the current study and the other literature mentioned above might be because of strongly enhanced adherence counseling and close monitoring of treatment. In addition, it may relate to differences in the treatment regimens used in this study compared with earlier studies. In the earlier studies, regimens like Stavudine, which had been omitted due to its devastating side effects, were included, and earlier discontinuation may be associated with it.

The quantity of ART pills taken per day was found to be an independent predictor of initial HAART discontinuation in the current study. When compared to patients who took one pill per day, those who took more than one pill per day were 4.1 times more likely to discontinue their initial regimen sooner. This finding is consistent with two studies conducted in the United States, in which patients treated with more than one pill per day were associated with a higher risk of discontinuation (HR = 3.44) [4], and pill burden, defined as taking more than one pill per day, was 1.44 times more hazardous to initial HAART discontinuation in the latter study [7]. Taking more than one medication each day can be considered as a surrogate for treatment complexity, which can be one possible cause of initial HAART regimen discontinuation. Although treatment complexity has been identified as a primary reason for HAART regimen discontinuation, few researches have examined its independent influence for HAART discontinuation [8–11]. The other argument is that therapy with more than one tablet daily might lead to poor adherence, which leads to poor outcomes in terms of virological suppression, CD4 increase, and opportunistic infection prevention, eventually leading to regimen discontinuation.

Those patients who had a low CD4 count at the initiation of ART have a higher risk of experiencing initial HAART discontinuation. Patients who had a CD4 count of < 100 cells/mm^3^, and those who had between 100 and 199 cells/mm^3^ were 2.6 times and 2.3 times at higher risk of experiencing initial HAART discontinuation, respectively, when compared with those with a CD4 count of ≥ 350 cells/mm^3^. This finding is similar to the studies done in West Africa, Kenya, the Netherlands, Canada, Peru, England (London) [2, 12–15]. Different explanations may exist for a lower CD4 count as a predictor of discontinuation of the initial HAART regimen. The first explanation is that a lower CD4 count is a marker of disease progression. This situation being a result of multiple viral replication cycles, may have led to the emergence of viral resistance resulting in less effective initial HAART. A second explanation may be that patients with lower CD4 counts are less compliant with HAART regimens, possibly due to more severe illness and intolerance to the therapeutic regimen [17].

Patients who had started HAART at baseline WHO clinical stages III and IV were nearly 2.6 times and 2.68 times at higher risk of discontinuing their initial regimen, respectively, as compared to those with WHO clinical stage I. This finding is similar to studies done in Switzerland and two Kenyan studies [1, 2, 18]. The possible explanation might be that the medical conditions of advanced HIV patients (WHO clinical stage III or IV) become worse because of various opportunistic infections. Due to those opportunistic infections, patients are likely to be on other medications, which might result in drug–drug interactions and side effects, which in turn reduce compliance and eventually drug discontinuation [19].

Those who had developed TB on the initial HAART regimen were nearly 2.3 times more at risk of discontinuing their initial regimen at any time, as compared to those who had not developed TB. This study was supported by a study done in India and Ethiopia [1, 5, 20], and other literature put it as one of the major reasons for regimen discontinuation [2, 16, 17]. Tuberculosis is one of the opportunistic infections that occur at any CD4 count in HIV patients. That might predict clinical failure if it occurs after 6 months of initiation of ART, which would subsequently indicate the need for initial HAART regimen discontinuation [13]. The other reason is the enzyme inducer nature of TB drugs. Especially Rifampicin induces cytochrome 450 enzymes that facilitate the metabolic activity of the liver, which is under the therapeutic level of ART drugs, especially Nevirapine (NVP), which results in viral resistance. This sounds like the need for regimen discontinuation when TB develops [19]. The other explanation might be that the anti-TB pill burden results in poor adherence and its common toxicity with ART drugs results in the need for discontinuation [4].

## Limitation of the study

A retrospective review of medical records may miss information that was not documented appropriately by the provider.

## Conclusion

The incidence of initial HAART regimen discontinuation was found to be high, and most of the discontinuations occurred after 1 year of the initiation of HAART, with a median survival time of 3.5 years. Having WHO clinical stage III or IV at the initiation of ART, having TB infection on the initial regimen, baseline CD4 count, and taking more than one ART pill per day were found to be predictors for time to initial HAART regimen discontinuation in the cohort of patients at Felege Hiwot comprehensive specialized hospital.

Every service point should give special attention to patients who have advanced disease (WHO clinical stages 3 and 4 and low baseline CD4 count). The test and treat strategy should be strengthened up to the community level to prevent the advancement of the disease.

ART service providers should initiate patients with the latest and most stable triple-fixed combination regimens that can be taken once daily. So Felege Hiwot comprehensive specialized hospital and other responsible stakeholders are expected to introduce alternative one-pill regimen preparations that lessen the pill burden.

TB and ART service providers should work in collaboration to reduce the tuberculosis burden on people living with HIV.

## Data Availability

The data used to support the findings of this study are available from the corresponding author upon request.

## Operational definitions

*Initial regimen discontinuation* is a switch or substitution (change or modification) of at least one drug from the original HAART regimen [4]. Regimen discontinuation, in this study as an event through the follow-up period, was ascertained retrospectively when the patients are recorded as discontinued their initial regimen and starts other HAART drugs.

*Event* is initial HAART discontinuation, which was ascertained retrospectively when the patient was recorded as discontinued the original regimen and started other HAART drugs within the duration of the follow-up period.

*Time to an event* is the time from the start of the initial HAART regimen to the date of discontinuation of that initial HAART regimen.

*Censored* Patients with the first date of loss to follow up, transfer out, death before the end of the follow-up period, and completed the follow-up period without developing the event were considered censored.

*Transfer out* a patient transferred to another health facility for care evidenced by their document.

*Survival* when the patient does not develop an event as evidenced by their clinical follow-up till the end of the study period.

*Survival time* The period that a patient stays in without developing an event after starting ART.

*Adherence* The extent to which a patient takes medications can be good, fair, or poor based on the patients’ ability to take ≥ 95%, 85–94%, and < 85% of the monthly refilled ART regimen, respectively evidenced by the document.

## Abbreviations

AHR: Adjusted hazard ratio
AIDS: Acquired immunodeficiency syndrome
ART: Antiretroviral treatment
CI: Confidence interval
HAART: Highly active antiretroviral therapy
IQR: Inter-quartile range
PY: Person years
TB: Tuberculosis
